# Is hidradenitis suppurativa more an autoinfection than pure autoinflammation?

**DOI:** 10.3389/fimmu.2026.1836916

**Published:** 2026-07-06

**Authors:** Maïa Delage, Olivier Join-Lambert, Snaigune Miskinyte, Alain Hovnanian, Aude Nassif

**Affiliations:** 1 Institut Pasteur, Centre Médical, Paris, France; 2 Univ de Caen Normandie, Univ Rouen Normandie, Institut national de la santé et de la recherche médicale (INSERM), Dynamique Microbienne associée aux Infections Urinaires et Respiratoires- Unité mixte de recherche (DYNAMICURE UMR) 1311, Centre hospitalier universitaire (CHU) Caen, Department of Infectious Agents, Caen, France; 3 Institut Imagine, INSERM UMR 1163, Laboratoire des Maladies Génétiques Cutanées, Paris, France

**Keywords:** autoinfection, hidradenitis suppurativa, inborn error of immunity, microbiology, pathophysiology, autoinflammatory disease, immune deficiency, host-microbiome disease

## Abstract

Hidradenitis suppurativa is currently considered an autoinflammatory disease. However, the cause of this inflammation remains unknown. Over the past 18 years, we have followed a cohort of 1,700 patients with hidradenitis suppurativa and identified, both in the literature and in our daily practice, several arguments suggesting that hidradenitis suppurativa represents a new concept of host–microbiome disease. This concept is based on an inborn error of immunity localized to a single organ—the skin—or, in special cases, to two or more organs. In this article, we detail the microbiological, immunologic, metabolomic, therapeutic, and genetic evidence supporting the view that HS should be redefined as an autoinfectious rather than a purely autoinflammatory disease.

## Introduction

Hidradenitis suppurativa (HS) is a complex polymorphous disease, currently classified as autoinflammatory (1). The source of this inflammation remains unknown, with only a few genes identified in a small percentage of patients, no metabolic pathway common to all HS cases, and no single treatment presently effective for all patients (2). Drawing on data from the medical literature and our clinical daily experience with a cohort of 1,700 patients with HS followed over the past 18 years, this article presents microbiologic, immunologic, metabolomic, therapeutic, and genetic findings and proposes a mechanism that may integrate these diverse data in this heterogeneous condition.

## Microbiologic anomalies in HS

At first, it should be noted that the only clinical manifestations of HS are abscesses, which are typically observed in infectious diseases.

In normal skin, bacteria are absent from the dermis. Several authors have reported the abnormal presence of bacteria within the dermis in HS lesions (3–8). Through prolonged cultures of ultrasound-guided biopsies, performed to avoid contamination from the skin surface, as well as bacterial metagenomic analyses, our team and others have confirmed that these are viable bacteria (4, 9). Our group and Naik et al. also demonstrated that the isolated flora differ according to disease severity (4, 8), consistent with the natural evolution of the disease. Indeed, the microbiology correlates with disease severity, with an increasing bacterial load and progressively greater diversity and antimicrobial resistance from Hurley stage I to stage III HS patients (4, 5, 8). This is characterized by a predominance of anaerobic bacteria, including *Prevotella* and *Porphyromonas*, as well as aero-anaerobes such as actinomycetes and the *Streptococcus milleri* group. In some mild cases of HS, a monomorphic flora consisting of either *Staphylococcus lugdunensis* or a single *Cutibacterium* species (most commonly *Cutibacterium avidum* or *Cutibacterium acnes*) can be isolated. In all the other cases, the flora is polymorphous, with a predominance of anaerobic bacteria (10).

In earlier studies, cultures from HS lesions isolated live bacteria in only 50% of cases (11). However, these studies lacked a transport medium for anaerobes (anaerobic bacteria die within 1 min in the presence of oxygen) and did not employ prolonged culture techniques (most bacteria from HS lesions require 4 to 7 days to grow, whereas standard cultures are maintained for only 48 h). Consequently, these incomplete studies concluded that the 50% of positive isolates represented contamination, while in reality, the negative cultures were largely attributable to inappropriate methodology—cultures that were too short and/or performed without a transport medium for anaerobes.

Most bacteria isolated from HS lesions belong to the patient’s own skin flora, with some secondary invaders. Under normal conditions, bacteria are located at the base of hair follicles or within follicles, but they do not reside in the dermis. In HS lesions, however, live bacteria are present in the dermis, accompanied by a marked infiltration of immune cells (12). If these dermal bacteria were merely innocent bystanders, what potent attractant could explain such a strong immune reaction with numerous immune actors in the dermis (13), if not the bacterial flora? Supporting this hypothesis, Williams et al. demonstrated a robust immune response in healthy keratinocytes following exposure to anaerobic Gram-negative bacteria, whereas keratinocytes from HS patients were not exposed to this bacterial stimulation (14).

In patients with inborn errors of immunity, polymicrobial infections often arise from their own self-flora as well as from community- or hospital-acquired flora (15). These bacteria are not considered contaminants, and appropriately adapted antibiotics (AB) usually cure such infections.

Similarly, although the flora abscesses are frequently polymicrobial (16, 17), these abscesses are treated as infections, with targeted antibiotics demonstrating therapeutic efficacy.

Many infections are associated with antibody production by the host in order to clear the infection. Interestingly, hypergammaglobulinemia is frequently observed in patients with HS and correlates with disease severity (18, 19). However, no study has yet investigated the presence of antibodies directed against the bacterial flora of HS lesions, which could represent disease markers and serve as a signature of the host’s defense reaction against bacteria that have become abnormally invasive.

Although not yet fully characterized, the microbiology of inflammatory diseases classically associated with HS shares common features with the flora observed in HS lesions:

- In dissecting cellulitis of the scalp, Brook reported a bacterial flora similar to that observed in HS lesions (20).
- In two patients presenting with pyoderma gangrenosum (PG) and HS, a flora resembling that of HS lesions was identified in PG lesions (21, 22).
- Interestingly, bacterial metagenomics has revealed a rich flora in the synovial membrane of patients with end-stage rheumatoid arthritis during surgical procedures for arthrodesis (23). Unexpectedly, this flora included *Porphyromonas*, *Prevotella*, and other organisms also present in HS lesions.
- In synovitis, severe acne, pustulosis, hyperostosis, and osteitis (SAPHO) syndrome—defined by the partial or complete association of these conditions—at least 39 references in PubMed describe the presence of *Cutibacterium acnes* in SAPHO lesions. A recent article even demonstrated complete and prolonged remission after combining antibiotic and surgical treatment (24).
- In Crohn’s disease, dysbiosis of gut flora has long been recognized, and Nucleotide-binding Oligomerization Domain containing 2 (*NOD2*) function—responsible for bacterial killing (25)—is impaired. This dysfunction may lead to uncontrolled proliferation and toxin production by commensal intestinal flora, which becomes abnormally invasive and produces intestinal fistulae. This mechanism is reminiscent of HS lesions.
- Behçet’disease, which can sometimes be associated with HS, has traditionally been described as presenting with sterile pseudo-folliculitis. However, in a series of 58 patients with Behçet’s disease, Hatemi (26), used prolonged cultures (rather than the usual 48-h cultures) and isolated several bacteria, including *Prevotella*, a predominant species typically recovered within 4 to 7 days from most HS lesions.
- In a patient with chronic acne fulminans + HS, bacterial metagenomics revealed that the flora from the acne fulminans lesion was highly similar to the flora usually observed in HS lesions (27).
- Granulomatous mastitis (GM), which can also be associated with HS, has been shown to harbor a bacterial flora resembling that of HS lesions (28–30).

In conclusion to this microbiological chapter, many diseases traditionally labeled as purely inflammatory may in fact involve microbial participation—a factor that has been overlooked for years due to inadequate techniques for microbial identification.

## Immunologic anomalies

Many immunologic anomalies have been identified in HS lesions that are considered self-explanatory:

- Various cell types have been implicated in HS lesions, including keratinocytes, B cells, plasma cells, M1 macrophages, T cells, fibroblasts, and MAIT cells (31–33). These cells may be attracted by bacteria abnormally present in the dermis; alternatively, another unknown trigger factor must be considered.
- Numerous cytokines and pathways, such as tumor necrosis factor (TNF), interleukin (IL)-1, IL-17, and IL-23, have been implicated in HS (13, 33). However, no single final pathway has been identified as common to all patients with HS.
- The same pathways are also implicated in inflammatory bowel diseases (IBD)—including TNF, IL-1, IL-6, IL-12/23, IL-17, and IFN (34)—as well as in rheumatologic inflammatory diseases, both of which can be associated with HS (35–38).
  Many immunologic anomalies have also been described in inborn errors of immunity, varying widely according to the type and degree of immune deficiency (39). If HS were fundamentally a heterogeneous group of distinct inborn errors of immunity with multiple different origins, this could explain the diversity of HS clinical forms as well as the range of immunologic and metabolic anomalies observed.

## Metabolomic anomalies

Our team observed a major increase in quinolinic acid and kynurenine in lesional biopsies from 20 patients with Hurley stage I HS, compared with healthy controls (40). These two compounds are produced through the tryptophan pathway. The tryptophan pathway has also been implicated in inflammatory rheumatologic diseases (41, 42) and in IBD (43). Moreover, the aryl hydrocarbon receptor pathway and the tryptophan pathway are closely interconnected (44), and their role in antimicrobial defense is suggested by LPS exposure (45). Taken together, these metabolic anomalies may impair antimicrobial defense, a disturbance also observed in inborn errors of immunity.

## Therapeutic data

At present, no single biotherapy achieves complete remission in all patients with HS, underscoring both the heterogeneity of the patient population and the challenges in treatment. Indeed, most biotherapies currently used in HS result in clinical improvement in approximately 50% of patients, while the remaining 50% are nonresponders (2).

In a cohort of kidney-transplanted patients receiving the immunosuppressant sirolimus, the usual prevalence of HS—approximately 1% in the general population—increased to 12% (46). This finding suggests that medically induced immunosuppression may aggravate HS in a manner reminiscent of inborn errors of immunity.

In the past, cultures from HS lesions were positive in only about 50% of cases and were incomplete, as they failed to isolate all HS-associated organisms that typically require up to a week to grow, rather than the usual 48 h. Consequently, antibiotics were prescribed in a largely empirical manner, since physicians could not rely on these noninformative cultures. These empiric treatments consisted mainly of cyclins, which yielded poor results (47). A plausible explanation for this failure is that cyclins target only a subset of organisms within the HS flora, resulting in incomplete treatment that addresses only part of the microbial community while potentially selecting for resistant strains.

Conversely, antibiotics targeted against HS-associated flora can induce complete remission (21, 27, 48–52). However, in our cohort of 1,700 HS patients receiving maintenance antibiotic therapy with cotrimoxazole or doxycycline, relapses during the past 17 years occurred only within scars and in the absence of triggering factors such as uncontrolled diabetes, use of systemic NSAIDs, systemic steroids, systemic immune suppressors, or significant weight gain (unpublished data). These findings suggest a role for biofilms, whose presence in chronic HS lesions has been confirmed by multiple authors (53–57). Given that very few medications can penetrate biofilms and that most antibiotics act primarily by inhibiting bacterial proliferation (thereby limiting their efficacy against dormant bacteria within biofilms), we subsequently implemented a systematic surgical excision of scars that had presented more than one relapse within 6 months after remission. Following these localized or extensive excisions, we obtained (and continue to observe) persistent remissions. The constant and persistent efficacy of surgery, which removes biofilms, further supports the role of biofilms in HS relapses.

If diseases classically associated with HS arise from the same microbiologic mechanism due to localized immune deficiency, they should benefit from targeted antibiotherapy.

- For example, a patient with chronic acne fulminans + HS, who had experienced constant flares for 3 years despite two lines of biotherapy combined with untargeted antibiotics, achieved remission through a prolonged HS-targeted antibiotic regimen. Remarkably, remission occurred simultaneously in both the acne fulminans lesions and the HS lesions (27).
- We reported a female patient with granulomatous mastitis and HS, who was treated for Hurley stage II HS with AB targeted against HS flora. She achieved remission of her granulomatous mastitis (48). Subsequently, 12 additional HS patients with granulomatous mastitis also obtained remission of both their HS and breast lesions; notably, four of these patients cancelled a scheduled mastectomy (58, 59).
- We also reported three patients with dissecting cellulitis of the scalp who achieved remission of their scalp lesions under an antibiotic regimen directed toward HS microbiology (60).
- We reported unexpected remissions of PG lesions in patients with both HS and PG treated with an AB strategy targeted against HS microbiology (21, 22).
- Interestingly and unexpectedly, we also observed (and continue to observe) a clear improvement in IBD symptoms in Hurley stage III Crohn + HS patients receiving ertapenem, including a significant decrease or complete disappearance of liquid and bloody stools and abdominal pain, despite our initial concern that ertapenem might worsen bloody diarrhea and abdominal pain (61, 62).

These therapeutic findings suggest that a precise and comprehensive microbiologic characterization of these conditions should be performed to determine whether these diseases share pathogenic mechanisms with HS.

The efficacy of targeted antibiotic therapy in HS and associated diseases suggests that an infectious component may contribute to the pathogenesis of these conditions. However, some authors have argued that antibiotics exert primarily anti-inflammatory effects in HS. Notably, ertapenem, currently recommended by North American HS guidelines as rescue therapy for severe and recalcitrant HS (63), has no known anti-inflammatory properties, whereas its antimicrobial spectrum closely matches the flora observed in Hurley stage III HS. Moreover, before we implemented targeted strategies based on Hurley’s severity score, which aligns with microbiological findings, we initially used empiric antibiotics that were not optimally targeted. Under these conditions, we observed that the same patient, after 6 weeks of incompletely targeted antibiotics, could achieve remission in some lesions but not others, even though the lesions were at the same stage of severity and located in different anatomical sites. For instance, remission could be observed in the left axilla and right groin, while persistent flares remained in the right axilla and left groin. If AB were acting solely through anti-inflammatory mechanisms, it would be difficult to explain why they appeared effective in some sites but not others within the same patient at the same time, particularly when microbiologic cultures revealed slight differences in flora among these areas (unpublished data).

## Genetic anomalies

Very few genes have been identified in HS. Mutations in gamma-secretase genes have been implicated in HS (64); however, the mechanisms leading to HS phenotypes remain unclear. Moreover, these mutations account for only about 5% of HS cases (65).

However, Colvin and Petukhova (66) recently reviewed mutations in inborn errors of immunity (IEI) in HS, and by adding our own identified IEI from our HS cohort and from the literature (67), a total of 50 genes have been described as mutated in association with HS. Indeed, HS diagnosis is often overlooked in IEI, as it is embedded within a more severe presentation of systemic infections, and diagnosis can be established only through careful reading of the case reports.

While the *PSTPIP1* gene regulates antimicrobial macrophage function (68), mutations in *PSTPIP1* have been reported in association with HS, pyoderma gangrenosum, severe acne, and suppurative hidradenitis (PASH), and pyogenic arthritis, pyoderma gangrenosum, severe acne, and suppurative hidradenitis (PAPASH) syndromes (69–72).

Several patients with HS (73, 74) and one patient with GM (75) were reported in association with a *NOD2* variant, while *NOD2* mutations, involved in antibacterial defense (25), are well-known in IBD patients (76).

At least seven patients with KID syndrome and HS were reported with a *GJB2* mutation, which represents an immune deficiency (77–82).

Seventy-four cases of PG from the literature were associated with IEI (83).

Moreover, inflammatory bowel diseases have been described in association with mutations in at least 69 genes of IEI (84–86), and inflammatory rheumatologic diseases have also been associated with mutations in 63 genes of IEI (87–89).

One family with a variant in *IRF2BP2*, a gene involved in a variable combined immune deficiency, was described by Palmroth. In this family, both a brother and a sister expressed only HS and no systemic infection, as is usually observed in patients with *IRF2BP2* mutations (90). We identified an *IRF2BP2* variant in one family with PASH family; the three adult patients carrying this variant have so far exhibited only cutaneous involvement, with no systemic infections in their past (91). Further investigations are required to determine why these patients do not exhibit systemic immune deficiencies but instead present with a skin-localized phenotype.

These associations of HS and other inflammatory diseases with IEI suggest that these conditions may rely, at least in part, on an immune skin deficiency responsible for abnormal and invasive bacterial proliferation from self-flora. This proliferation perpetuates immunologic activation by the host, who attempts to clear the resulting “autoinfection”. However, because of the underlying skin IEI, the host is unable to eradicate this flora, and the immune system continues to fight the bacterial invasion, thereby sustaining chronic inflammation. Such inflammation can only be resolved through bacterial removal (either definitively by surgery or temporarily by antibiotics).

Based on these data and clinical observations, we propose that HS should be redefined as at least partly autoinfectious as well as autoinflammatory.

## Conclusion

Rethinking HS as many different inborn errors of immunity localized to the skin or, in rare cases, associated with systemic immune deficiencies, could explain the diversity of clinical, immunologic/metabolic, and microbiologic pictures responsible for different responses to various treatments, including biotherapies and antibiotics. These considerations may help better understand the mechanisms involved in the disease and provide arguments for combining strategies, for instance, associating biotherapies and targeted antibiotics according to severity to prepare patients for surgery and obtain a more definitive remission.

## Data Availability

The original contributions presented in the study are included in the article/supplementary material. Further inquiries can be directed to the corresponding author.
